# The atypical antipsychotics and sexual dysfunction: a pharmacovigilance-pharmacodynamic study

**DOI:** 10.3389/fphar.2024.1423075

**Published:** 2024-07-09

**Authors:** Yu Cheng, Youjun Chen, Xue Zhao, Fan Mou, Wanying Wang, Ruiyi Qian, Jingjing Huang, Huafang Li, Qingqing Xu, Shunying Yu

**Affiliations:** ^1^ Shanghai Mental Health Center, Shanghai Jiao Tong University School of Medicine, Shanghai, China; ^2^ Shanghai Ninth People’s Hospital, College of Stomatology, Shanghai Jiao Tong University School of Medicine, Shanghai, China

**Keywords:** atypical antipsychotics, sexual dysfunction, real-world data, disproportionality analysis, safety signal

## Abstract

**Background:**

Atypical antipsychotics (AAPs)-induced sexual dysfunction (SD) is a frequent issue in clinical practice, often underestimated by clinicians and not extensively researched. The current study aimed to quantify the strength of association between the use of different AAPs and SD using real-world data from the FDA Adverse Event Reporting System (FAERS), as well as investigate the receptor mechanisms that are involved.

**Methods:**

Data from the FAERS database from the first quarter of 2004 to the third quarter of 2023 were queried through OpenVigil 2.1. Disproportionality analysis was estimated using the reporting odds ratio (ROR) and information component (IC) methods, and linear regression was used to investigate the relationship between ROR and receptor occupancy which was estimated using *in vitro* receptor binding profiles.

**Results:**

Our analysis yielded 4839 reports that co-mentioned AAP and SD events, and the findings revealed statistical associations between 12 AAPs and SD. The highest signal value was identified for iloperidone reporting retrograde ejaculation with iloperidone (ROR = 832.09, ROR_025_ = 552.77; IC = 9.58, IC_025_ = 6.36), followed by compulsive sexual behavior with aripiprazole (ROR = 533.02, ROR_025_ = 435.90; IC = 7.30, IC_025_ = 5.97), and psychosexual disorder for aripiprazole (ROR = 145.80, ROR_025_ = 109.57; IC_025_ = 6.47, IC025 = 4.86). Different characteristics of the SD side effects in each AAPs were discovered after further data mining. Regression analysis revealed potential effects for receptor occupancy of D2, D3, and 5-HT1A receptors on ROR. However, no significant correlation persisted following sensitivity analyses.

**Conclusion:**

This is the first study to investigate the AAP-SD associations by using FAERS. In this study, we report for the first time a significant association between aripiprazole and SD based on real-world data. The study suggests that different AAPs have varying levels of association with SD, and the D2, D3, and 5-HT1A receptor occupancy may contribute to potential mechanisms. The findings of this study warrant further validation of more studies and clinical causality assessment.

## 1 Introduction

Antipsychotic medications are a critical component in managing schizophrenia and other psychotic disorders (Levenson, 2024). The introduction of atypical antipsychotics (AAPs) has led to a significant rise in their prescription across numerous countries, both in FDA-approved applications and off-label uses (Verdoux et al., 2010; Hermes et al., 2013). However, the increasing use of AAPs has also highlighted concerns over their safety profiles. The commonly reported adverse events (AEs) of AAPs, such as weight gain, metabolic changes, sedation, can lead to medication discontinuation and hinder the desired therapeutic outcomes. Moreover, sexual dysfunction (SD) is a common side effect of AAPs that can significantly diminish life quality, and negatively affect medication adherence, but it is often underestimated in clinical practice (Clayton et al., 2016). SD encompasses persistent and disruptive issues that can affect any phase of the sexual response cycle in both men and women. These symptoms may manifest as problems with penile erection, lubrication, orgasm, libido, retrograde ejaculation, sexual arousal, or general sexual satisfaction.

The occurrence of antipsychotics-induced SD varies greatly across different studies (Bobes et al., 2003; Montejo et al., 2010; Serretti and Chiesa, 2011). The significant difference in these rates could be influenced by many factors, including the use of various assessment techniques, sub-optimal communication about sexuality between patients and clinicians, shame, cultural difficulties and lack of interest by healthcare professionals, insufficient knowledge and awareness of sexually-related adverse events, and more (Gordijn et al., 2019). A study conducted on 750 Italian psychiatrists revealed that a mere 3% of them reported regularly assessing sexuality using psychometric tests (Monteleone et al., 2019). Another cross-sectional study found that a vast majority (73.2%) of healthcare professionals did not routinely inquire about sexual problems during clinical consultations and admitted to a lack of expertise (Tharoor et al., 2015). For some patients, the subjective burden of SD can be as high as the burden of the disease itself (de Boer et al., 2015).

Antipsychotics are commonly used for treating mental disorders, but there are concerns regarding their impact on sexual functioning. Comparing antipsychotics can be challenging as there is limited evidence available from clinical trials and systematic reviews. According to a meta-analysis (Serretti and Chiesa, 2011), there are notable variations in the incidence rates among different antipsychotics regarding total SD. Quetiapine, ziprasidone, and aripiprazole were associated with relatively low SD rates (16%–27%), whereas olanzapine, risperidone, and clozapine were associated with higher SD rates (40%–60%). Despite the aforementioned evidence, it remains uncertain which AAP has a greater impact on sexual functioning when compared to another. The exact mechanisms underlying antipsychotic-induced SD have not been fully understood. Researchers have explored several receptor mechanisms: dopamine receptor antagonism reduces libido and impairs arousal and orgasm by inhibiting motivation and reward or elevating prolactin levels; histamine receptor antagonism impairs arousal by increasing sedation. Additionally, cholinergic receptor antagonism or alpha-adrenergic receptor antagonism might cause erectile dysfunction by reducing peripheral vasodilation (Knegtering et al., 2003; Haddad and Wieck, 2004; La Torre et al., 2013). These findings highlight the need to consider the impact of antipsychotics on sexual functioning while prescribing them. It is essential to understand the impact of antipsychotics on sexual functioning to help patients achieve better mental and sexual health outcomes.

The available evidence of SD induced by AAPs is mainly affected by the insufficient epidemiological data and methodological limitations of related research. Hence, using data from an enlarged real-world source might provide a better approach. Post-marketing data, in particular, is poised to significantly illuminate this matter due to its extensive collection of information from individuals undergoing drug treatments, thus furnishing more evidence of this correlation. The United States Food and Drug Administration (FDA) Adverse Event Reporting System (FAERS) contains millions of real-world reports from various sources, and it has been widely used in pharmacovigilance studies and drug safety evaluations. Our aim is to quantify the strength of association between different AAPs and SD-related AEs. We also investigate whether the safety signal of reporting SD is affected by the neurotransmitter receptor occupancy. Our findings will provide better insight into drug safety evaluations and the correlation between AAPs and SD.

## 2 Materials and methods

### 2.1 Data source

FAERS database is a publicly available global spontaneous reporting system, gathering reports from healthcare professionals, patients, manufacturers, etc. The suspected AEs, which can be identified by preferred terms (PTs), as coded by the Medical Dictionary for Regulatory Activities (MedDRA) (https://www.meddra.org/). The hierarchical structure of MedDRA enables PTs to be grouped into higher levels, including High-Level Terms (HLTs), High-Level Group Terms (HLGTs), and System Organ Classes (SOCs), which provides flexibility in retrieving AEs of interest.

For the current analysis, FAERS reports from the first quarter of 2004 to the third quarter of 2023 were queried by using the online tool OpenVigil 2.1 (https://openvigil.sourceforge.net/). OpenVigil is an innovative web-based pharmacovigilance analysis tool designed to access pharmacovigilance data from the FAERS database (Bohm et al., 2012). It operates on cleaned data, which includes verified and normalized drug names, and supports data extraction, cleaning, mining, and analysis of the FAERS database.

### 2.2 Data collection

The AAPs investigated here were “amisulpride,” “aripiprazole,” “asenapine,” “brexpiprazole,” “cariprazine,” “clozapine,” “iloperidone,” “loxapine,” “lurasidone,” “olanzapine,” “paliperidone,” “quetiapine,” “risperidone,” and “ziprasidone”. To minimize the impact of confounding variables, we exclusively incorporated reports wherein the target medication was designated as the “primary suspect” (PS). The PS drug refers to situations in which the reporting individual identifies the drug as the most likely causative factor for the particular AE under consideration. To comprehensively capture AEs of interest, PTs within the HLGT = “Sexual function and fertility disorders” and HLGT = “Sexual dysfunctions, disturbances and gender identity disorders” in MedDRA (version 26.0) were considered for the subsequent analysis. All PTs related to different SD manifestations included in the analysis are listed in Supplementary Table S2. It is important to clarify that there is a PT term “sexual dysfunction” under the HLGT “Sexual function and fertility disorders.” It is one basic term among the various PT terms related to SD manifestations discussed in this paper, without a specific definition. This should be distinguished from the SD referenced throughout the study. To better characterize their safety profile, we classified reports with positive signals as “hypersexuality”, “hyposexuality”, “erectile dysfunction”, and “ejaculatory dysfunction” according to the main presenting symptoms of SD proposed by Yang and Wang (2016). The specific PT terms assigned to the four subgroups are presented in Supplementary Table S1.

The drug-receptor interactions were quantified based on the receptor occupancy theory. Ten receptors: serotonin receptors (5-HT1A, 5-HT2A, 5-HT2C, and 5-HT7), adrenergic receptor alpha1/2 (irrespective of subtype), muscarinic receptors (regardless of subtype), dopamine receptors D2 and D3, as well as histamine receptor H1 were involved in the quantification. The occupancy (%) is calculated using the formula: occupancy (%) = 100 * (C_U_/(K_i_ + C_U_), where C_U_ (nM) is the unbound drug concentration in the blood and Ki is the inhibition constant of the drug (Kenakin, 2004). C_U_ is calculated by the formula: C_U_ = 1000 * F_U_ * C_T_/MW. F_U_ is the unbound drug fraction extracted from the Drugbank (https://go.drugbank.com/). C_T_ represents the blood drug concentration, which was estimated based on the upper threshold of the recommended therapeutic drug levels of each AAPs (Hiemke et al., 2018). MW is the molecular weight extracted from the International Union of Basic and Clinical Pharmacology (IUPHAR) and the British Pharmacology Society (BPS) dataset (Harding et al., 2018). The Ki values were extracted from the psychoactive drug screening program database (Roth et al., 2000), and unavailable Ki data was from the IUPHAR/BPS dataset. The pharmacodynamic data used in the present analysis were mainly drawn from the study conducted by Cepaityte et al. (2021). Receptor occupancies are presented in Figure 2.

To conduct sensitivity analyses, we obtained information on drug activities, including full agonist, partial agonist, antagonist, inverse agonist, and unspecified for each AAP from DrugBank.

### 2.3 Data analysis

Cases were represented by AEs of interest in which AAPs were assigned as “PS”, and non-cases were all other reports of FAERS during the examined period. To be included in the final analysis, the count for any selected PT needed to be greater than 3. Reporting odds ratios (ROR) and information component (IC) methods were employed to detect the safety signals. The ROR held significance when the lower limit of its 95% confidence interval (ROR_025_) surpassed 1; similarly, the IC was deemed significant when its lower limit (IC_025_) exceeded 0 (Bate and Evans, 2009; Harpaz et al., 2013). We applied linear regression models to investigate potential associations between the median ROR of each AAP under examination and receptor occupancy. In this analysis, the median ROR of individual AAP served as the dependent variable, while the estimated receptor occupancy was considered the independent variable. Only receptors with at least three occupancy data were included.

The following sensitivity analyses were performed on the primary outcome ROR: 1) in May 2016, FDA issued a relevant warning for aripiprazole-induced impulse-control disorders (ICDs) including also “compulsive sexual behaviors,” “hypersexuality,” “excessive masturbation” and “libido increase” that queried as SD-related PT terms in this study. What’s more, ICDs are also already listed in the summary of product characteristics of brexpiprazole in 2018. The warning may have impacted the reporting of related SD AEs not only for the concerned drugs, but also for the other AAPs. To account for potential notoriety bias by relevant FDA warning, reports before and after the FDA warning were analyzed; 2) to minimize the impact of event-related biases (i.e., high-signal with a small number of reports), we excluded PTs with fewer than 500 total reports.

Also, sensitivity analyses were conducted on the linear regression model: 1) drugs can interact with receptors in various ways, being classified as agonists, antagonists, partial agonists, or inverse agonists. Consequently, an agonist and an antagonist with identical affinity can produce opposite effects. To address this, we reversed the receptor occupancy sign for antagonists and inverse agonists. Drugs for which we could not retrieve activity were excluded from these models; 2) calculating receptor occupancy requires multiple data, which often comes from varied sources. This variability can lead to inconsistencies and potentially affect the reliability of regression models. To minimize the impact of data discrepancies, we use pKi values (the logarithm with the base 10 of Ki) instead; 3) given that PTs related to ICD might influence the signal values, particularly for aripiprazole, brexpiprazole, and cariprazine, we repeated sensitivity analysis 1) after excluding these PTs. A relationship was considered robust if it was confirmed by at least two different sensitivity analyses.

Microsoft Excel 20.0, GraphPad Prism 9, R version 4.3.0 and SPSS 23.0 were used to perform all analyses.

### 2.4 Causality assessment

Austin Bradford-Hill criteria, initially intended for linking environmental factors to disease causation, have been broadly applied in epidemiology and are also relevant to pharmacovigilance (Shakir and Layton, 2002). Using the adapted criteria that integrate existing evidence and disproportionality (Raschi et al., 2021), we systematically gathered evidence for AAP-induced SD. These criteria included considerations of biological plausibility, strength, consistency, specificity, coherence, and analogy.

## 3 Results

### 3.1 Descriptive analysis

The final analysis included 4839 reports, encompassing a total of 12 atypical antipsychotics. Of the detected AAPs, aripiprazole comprised the most cases (33.7%), followed by risperidone (17.2%) and quetiapine (16.9%). The mean patient age was 35.1 years (standard deviation 13.4). SD reports showed a significantly higher proportion of younger individuals (52.9% vs 1.4%) and men (67.2%vs 23.3%). More than 50% of AE reports were submitted from the United States, and 47.3% were from other countries or were unspecified. Regarding the indication for use, schizophrenia and bipolar disorders were more frequently represented in AAP-related SD reports. Throughout the study period, 3.7% of the cases had fatal outcomes, while 21.5% reported hospitalization. The number of submitted reports related to AAP-induced SD has shown an increasing trend every 5 years. Reports numbered 404 from 2004 to 2008, 996 from 2009 to 2013, 1488 from 2014 to 2018, and 1951 from 2019 to 2023. 24.2% of the reports provided dosage information, with the average daily dose for all medications falling within the therapeutic range. The detailed characteristics of analyzed cases are listed in Table 1.

**TABLE 1:** Event characteristics as reported to FAERS for evaluated atypical antipsychotics-sexual dysfunction adverse events.

Characteristics	Aripiprazole	Asenapine	Brexpiprazole	Cariprazine	Clozapine	Iloperidone	Lurasidone	Olanzapine	Paliperidone	Quetiapine	Risperidone	Ziprasidone
Age distribution												
Adult n (%)	691 (42.3)	11 (29.7)	13 (36.1)	10 (30.3)	120 (81.1)	37 (43.0)	32 (33.0)	387 (70.9)	192 (43.7)	574 (70.0)	432 (51.9)	61 (46.2)
18-40	475 (29.1)	8 (21.6)	5 (13.9)	8 (24.2)	105 (70.9)	25 (29.1)	23 (23.7)	271 (49.6)	139 (31.7)	327 (39.9)	302 (36.3)	41 (31.1)
41-65	216 (13.2)	3 (8.1)	8 (22.2)	2 (6.1)	15 (10.1)	12 (14.0)	9 (9.3)	116 (21.2)	53 (12.1)	247 (30.1)	130 (15.6)	20 (15.2)
Elderly >65 n (%)	17 (1.0)	0 (0.0)	1 (2.8)	0 (0.0)	0 (0.0)	0 (0.0)	0 (0.0)	9 (1.6)	3 (0.7)	25 (3.0)	15 (1.8)	0 (0.0)
*p-value*	0.000	1.000	0.488	1.000	0.000	0.152	0.069	0.000	0.015	0.000	0.000	0.051
Others and Unknown	924 (56.6)	26 (70.3)	22 (61.1)	23 (69.7)	28 (18.9)	49 (57.0)	65 (67.0)	150 (27.5)	244 (55.6)	221 (27.0)	386 (46.3)	71 (53.8)
Mean Age (SD)	34.4 (13.6)	37.0 (7.7)	46.5 (11.2)	36.5 (6.9)	29.1 (8.4)	34.2 (10.5)	32.1 (11.0)	35.3 (12.9)	33.8 (12.1)	37.7 (14.2)	34.5 (13.8)	33.9 (13.8)
Gender n (%)												
Female	522 (32.0)	20 (54.1)	21 (58.3)	11 (33.3)	72 (48.6)	1 (1.2)	36 (37.1)	131 (24.0)	32 (7.3)	170 (20.7)	96 (11.5)	16 (12.1)
Male	918 (56.3)	15 (40.5)	11 (30.6)	19 (57.6)	71 (48.0)	79 (91.9)	35 (36.1)	391 (71.6)	355 (80.9)	606 (73.9)	651 (78.2)	99 (75.0)
*p-value*	0.000*	0.507	0.725	0.001*	0.004*	0.000*	0.001*	0.000*	0.000*	0.000*	0.000*	0.000*
Unknown	192 (11.8)	2 (5.4)	4 (11.1)	3 (9.1)	5 (3.4)	6 (7.0)	26 (26.8)	24 (4.4)	52 (11.8)	44 (5.4)	86 (10.3)	17 (12.9)
Country n (%)												
United States	984 (60.3)	16 (43.2)	34 (94.4)	25 (75.8)	13 (8.8)	85 (98.8)	80 (82.5)	130 (23.8)	339 (77.2)	382 (46.6)	355 (42.6)	105 (79.5)
Others and Unknown	648 (39.7)	21 (56.8)	2 (5.6)	8 (24.2)	135 (91.2)	1 (1.2)	17 (17.5)	416 (76.2)	100 (22.8)	438 (53.4)	478 (57.4)	27 (20.5)
Indication n (%)												
Psychotic disorder	547 (33.5)	20 (54.1)	2 (5.6)	5 (15.2)	119 (80.4)	31 (36.0)	21 (21.6)	234 (42.9)	178 (40.5)	175 (21.3)	351 (42.1)	24 (18.2)
Bipolar disorder	331 (20.3)	3 (8.1)	2 (5.6)	9 (27.3)	3 (2.0)	4 (4.7)	29 (29.9)	50 (9.2)	27 (6.2)	120 (14.6)	58 (7.0)	18 (13.6)
Others and Unknown	754 (46.2)	14 (37.8)	32 (88.9)	19 (57.6)	26 (17.6)	51 (59.3)	47 (48.5)	262 (48.0)	234 (53.3)	525 (64.0)	424 (50.9)	90 (68.2)
Outcome n (%)												
Death/Life threatening	52 (3.2)	3 (8.1)	0 (0.0)	0 (0.0)	2 (1.4)	3 (3.5)	2 (2.1)	33 (6.0)	12 (2.7)	25 (3.0)	45 (5.4)	2 (1.5)
Hospitalization	490 (30.0)	4 (10.8)	0 (0.0)	5 (15.2)	29 (19.6)	14 (16.3)	4 (4.1)	113 (20.7)	37 (8.4)	153 (18.7)	166 (19.9)	26 (19.7)
Other and Unknown	1090 (66.8)	30 (81.8)	36 (100.0)	28 (84.8)	117 (79.1)	69 (80.2)	91 (93.8)	400 (73.3)	390 (88.8)	642 (78.3)	622 (74.7)	104 (78.8)
Year n (%)												
2004-2008	67 (4.1)	0 (0.0)	0 (0.0)	0 (0.0)	7 (4.7)	0 (0.0)	0 (0.0)	50 (9.2)	29 (6.6)	107 (13.0)	105 (12.6)	39 (29.5)
2009-2013	128 (7.8)	13 (35.1)	0 (0.0)	0 (0.0)	23 (15.5)	53 (61.6)	0 (0.0)	71 (13.0)	163 (37.1)	271 (33.0)	209 (25.1)	65 (49.2)
2014-2018	761 (46.6)	3 (8.1)	7 (19.4)	3 (9.1)	13 (8.8)	16 (18.6)	46 (47.4)	179 (32.8)	40 (9.1)	129 (15.7)	274 (32.9)	17 (12.9)
2019-2023	676 (41.4)	21 (56.8)	29 (80.6)	30 (90.9)	105 (70.9)	17 (19.8)	51 (52.6)	246 (45.1)	207 (47.2)	313 (38.2)	245 (29.4)	11 (8.3)
Mean dose, mg (N with available data)	9.63 (398)	14.2 (12)	2.15(17)	2.82 (17)	268.9 (33)	11.5(35)	39.8 (32)	11.5 (240)	12 (1)	295.1 (190)	3.3(144)	110.2 (54)

### 3.2 Disproportional analysis

Signal detection results for specific PT were illustrated in Supplementary Table S2. The current safety signal analyses showed that SD was significantly associated with 12 AAPs, which exhibited distinct AE profiles. Aripiprazole was identified with the widest spectrum of SD-related events with 25 PTs, followed by paliperidone (N = 19), quetiapine (N = 19) and risperidone (N = 18). The largest effect size in this estimation was observed for retrograde ejaculation with iloperidone (ROR = 832.09, ROR_025_ = 552.77; IC = 9.58, IC_025_ = 6.36), followed by compulsive sexual behavior with aripiprazole (ROR = 533.02, ROR_025_ = 435.90; IC = 7.30, IC_025_ = 5.97), and psychosexual disorder for aripiprazole (ROR = 145.80, ROR_025_ = 109.57; IC_025_ = 6.47, IC025 = 4.86).

**FIGURE 1:**
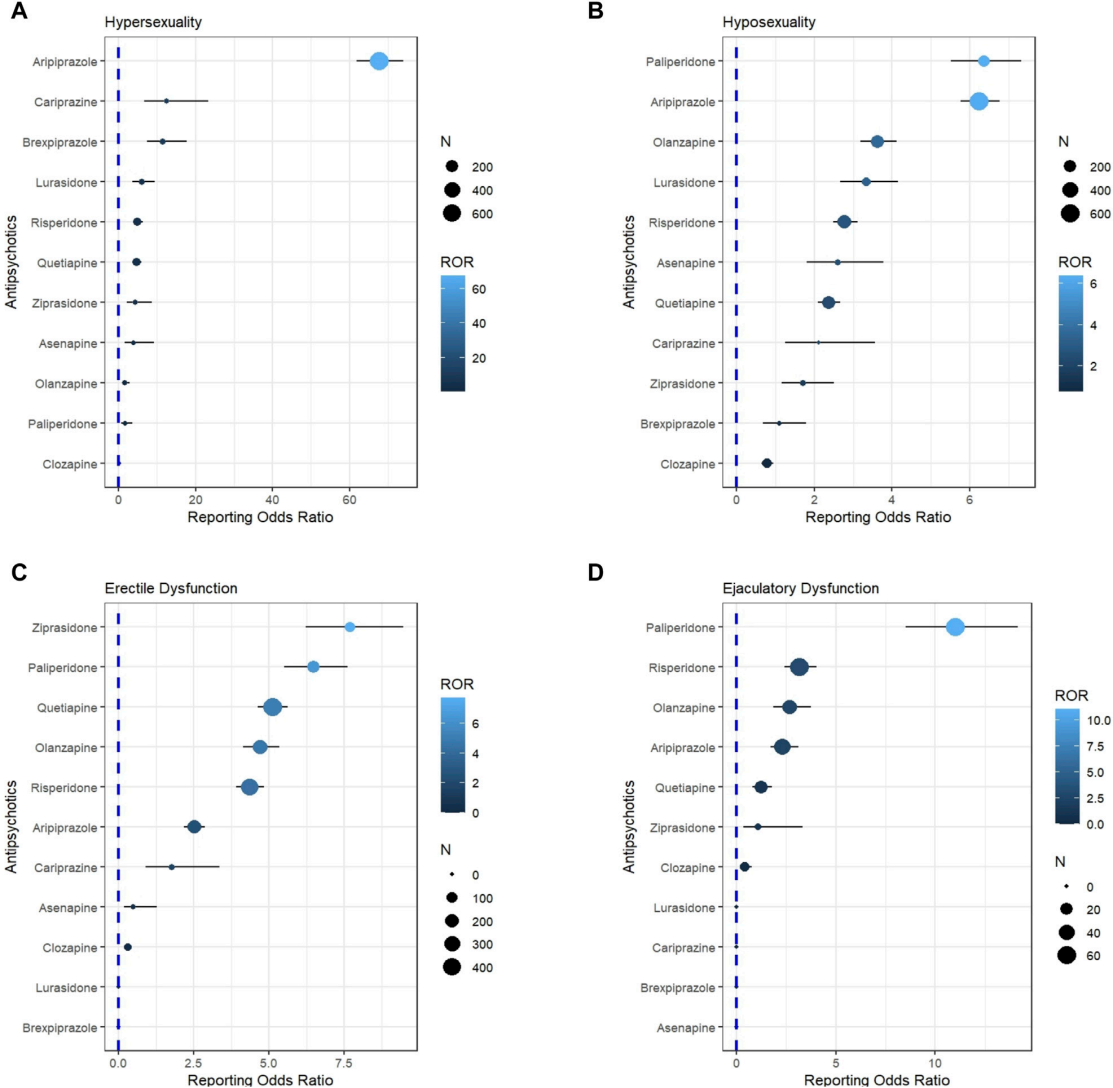
Detected signal associations between atypical antipsychotics and four subgroups of sexual dysfunction manifestations. **(A)** Represents the association between AAPs and hypersexuality **(B)** represents the association between AAPs and hyposexuality **(C)** represents the association between AAPs and erectile dysfunction **(D)** represents the association between AAPs and ejaculation dysfunction.

It is worth noting that the number of iloperidone reports during the study period was only 845, considerably fewer than the reports for other AAPs, which could potentially introduce reporting bias and affect the study results. Herein, iloperidone was subsequently excluded from the subgroup analysis and receptor mechanism analysis. Regarding the subgroup analyses of main symptoms, aripiprazole exhibited the most robust signal for hypersexuality (ROR = 67.65). Paliperidone exhibited the highest ROR value for hyposexuality (ROR = 6.35), and ejaculatory dysfunction (ROR = 10.99). Ziprasidone was observed with the highest signal for erectile dysfunction (ROR = 7.69) (Figure 1). In sensitivity analyses for ROR, we found that although most AAPs (except asenapine, cariprazine, clozapine, and lurasidone) were significantly associated with SD before the FDA warning, the number of reports and signal strength increased significantly after the warning, particularly for aripiprazole and cariprazine. The notoriety bias may have influenced non-warning AAPs to some extent. In sensitivity analysis 2) for ROR, after excluding PTs with a small number of total reports, all AAPs remained significantly associated with SD (Table 2).

**TABLE 2:** Sensitivity analyses for disproportionality approach.

	Aripiprazole	Asenapine	Brexpiprazole	Cariprazine	Clozapine	Lurasidone	Olanzapine	Paliperidone	Quetiapine	Risperidone	Ziprasidone
Restricted to the 2004.01–2015.05 period N, ROR (95%CI)	268, 1.92 (1.70–2.17)	13, 1.66 (0.96–2.86)	4, 5.33 (1.99–14.24)	0	35, 1.34 (0.96–1.86)	3, 1.47 (0.47–4.57)	187, 1.95 (1.68–2.25)	207, 4.70 (4.09–5.40)	450, 2.38 (2.17–2.61)	495, 3.52 (3.22–3.85)	118, 5.30 (4.41–6.36)
Restricted to the 2016.06–2023.09 period N, ROR (95%CI)	1370, 17.99 (17.02–19.03)	24, 26.55 (17.69–39.86)	32, 6.21 (4.39–8.80)	33, 4.86 (3.45–6.85)	113, 3.83 (3.17–4.61)	94, 5.72 (4.66–7.01)	360, 9.21 (8.29–10.24)	232, 10.75 (9.43–12.26)	375, 6.58 (5.94–7.30)	341, 4.19 (3.76–4.66)	16, 8.51 (5.19–13.94)
Exclude potentially influenced PT ^a^ N, ROR (95%CI)	1139, 6.01 (5.66–6.38)	37, 5.29 (3.83–7.31)	30, 4.42 (3.09–6.33)	33, 3.57 (2.54–5.04)	135, 2.50 (2.11–2.97)	94, 4.23 (3.45–5.19)	497, 4.35 (3.98–4.76)	390, 6.92 (6.26–7.66)	744, 3.81 (3.54–4.10)	758, 3.95 (3.68–4.25)	132, 7.48 (6.30–8.89)

N, the number of reports; ROR, reporting odds ratio; CI, confidence interval.

^a^ Excluded were the Preferred Terms (PTs) with fewer than 500 reports in the FAERS database during the study period.

### 3.3 Association analysis between ROR signal and receptor occupancy

**FIGURE 2:**
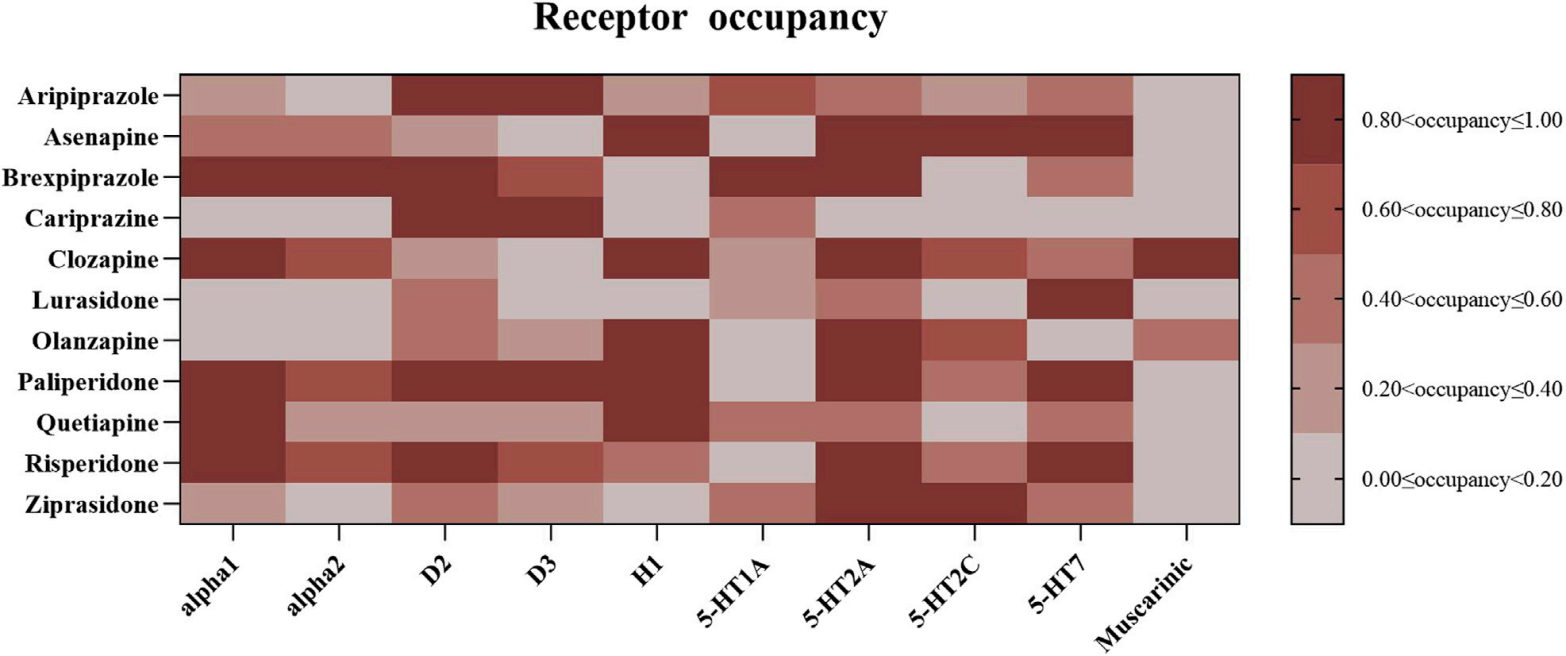
Occupancy of atypical antipsychotics to neurotransmitter receptors.

**FIGURE 3:**
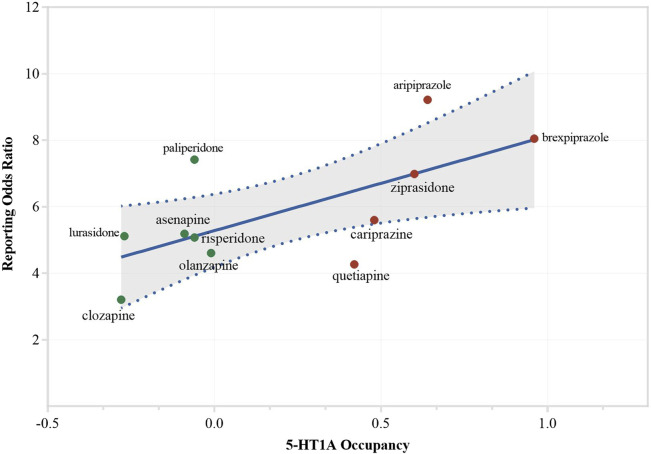
Association between activity on 5-HT1A and reporting of sexual dysfunction. Sensitivity analysis (1), considering different activities. Drugs were color-coded to show their activity: red indicates agonists (including partial agonists), green indicates antagonists.

Receptor occupancy was calculated for the investigated AAPs (Figure 2). To explore the relationship between receptor occupancy and the disproportionate reporting of AAP-related SD, we applied univariate linear regression models and conducted three sensitivity analyses. The primary analysis identified three significant correlations between receptor occupancy and SD reporting (median ROR): D2-receptor (β = 4.516, *p* = 0.014, *R* ^2^ = 0.505), D3-receptor (β = 3.330, *p* = 0.037, *R* ^2^ = 0.399), and muscarinic-receptor (β = −4.112, *p* = 0.042, *R* ^2^ = 0.385) (see Table 3; Supplementary Figure S1). However, in the sensitivity analyses, no receptor showed significance across two tests. When accounting for different receptor activities, only the 5-HT1A receptor exhibited a significant association in the regression model (β = 2.847, *p* = 0.026, *R* ^2^ = 0.441) (see Table 3; Figure 3). After excluding ICD-related PTs, none of the receptors retained significance.

**TABLE 3:** Linear regression models between reporting odds ratio and receptor occupancy.

Receptor	Main analysis			Sensitivity analysis 1			Sensitivity analysis 2			Sensitivity analysis 3		
	β	*p*-value	*R* ^2^	β	*p*-value	*R* ^2^	β	*p*-value	*R* ^2^	β	*p*-value	*R* ^2^
Alpha1	−0.424	0.791	0.008	0.824	0.653	0.026	−0.559	0.428	0.071	0.493	0.787	0.010
Alpha2	0.209	0.911	0.001	0.409	0.863	0.005	−0.412	0.472	0.059	1.985	0.350	0.125
D2	4.516	0.014*	0.505	1.220	0.146	0.219	−1.274	0.016*	0.494	−0.504	0.563	0.039
D3	3.330	0.037*	0.399	0.942	0.350	0.125	−1.139	0.021*	0.465	−0.735	0.489	0.071
H1	−2.420	0.088	0.289	2.420	0.088	0.289	0.518	0.414	0.075	0.475	0.754	0.011
5-HT1A	3.393	0.070	0.319	2.847	0.026*	0.441	−0.746	0.089	0.288	0.219	0.880	0.003
5-HT2A	−0.364	0.870	0.003	0.364	0.870	0.003	−0.397	0.523	0.047	−0.837	0.702	0.017
5-HT2C	−1.304	0.451	0.064	2.072	0.168	0.252	−0.113	0.816	0.006	1.592	0.341	0.130
5-HT7	0.068	0.972	0.000	2.715	0.100	0.385	−0.455	0.416	0.075	1.765	0.305	0.173
Muscarinic	−4.112	0.042*	0.385	−1.456	0.569	0.119	0.890	0.115	0.253	−1.753	0.203	0.467

*p-value is significant.

### 3.4 Bradford hill criteria

Overall, based on the disproportionate signal values, sensitivity analysis, and support from existing studies, most of the criteria have been met, including strength, biological plausibility and experimental support, coherence, consistency, and analogy. However, due to the inability of OpenVigil 2.1 to obtain data related to the assessment of dechallenge and rechallenge, temporality cannot be clearly determined. Additionally, the current data and relevant studies do not allow for a determination of whether biological gradient and specificity are met (Table 4). To some extent, a likely causal association may exist between AAP use and SD.

**TABLE 4:** Causality assessment of sexual dysfunction (SD) with atypical antipsychotics (AAPs) based on Bradford Hill criteria.

Criterium	Findings	Criterium fulfilled
Strength of the association	We employed the ROR to assess the strength of the association between AAPs and SD. Although ROR is not a direct measure of risk, its magnitude indicates a strong signal. All the examined AAPs show significant association signals	**√**
Biological plausibility and experimental support	Antipsychotic drugs impact various neurotransmitter systems, such as cholinergic, adrenergic, histaminergic, and dopaminergic pathways, albeit to varying degrees depending on the specific drug. This diverse receptor interaction can lead to sexual dysfunction: histamine receptor antagonism impedes arousal; dopamine receptor antagonism affects libido and arousal; cholinergic and alpha-adrenergic receptor antagonism contribute to erectile dysfunction and decreased lubrication, etc. Some clinical studies also provide evidence that atypical antipsychotics are associated with a substantial impairment of sexual functioning	**√**
Coherence	Case reports, clinical studies, and animal research all indicate that the use of AAP may induce SD, although there may be inconsistencies in risk among different studies regarding various medications	**√**
Consistency	Results of disproportionality approaches were consistent across different sensitivity analyses	**√**
Specificity	There is no precise definition of SD, and the PTs included in the analysis may be incomplete or may not be classified as SD from different study perspectives. Additionally, due to data limitations in the FAERS database, it is impossible to determine the specificity of the association between the target drug and the target adverse events	**?**
Biological gradient	The available data does not establish a precise relationship between dosage or duration and adverse reactions. Despite missing data, SD do occur within the therapeutic dose range	**?**
Temporality	Due to the unavailability of time-to-onset data, an analysis of this factor cannot be performed	**?**
Analogy	Atypical antipsychotic drugs are recognized for their role in inducing SD. Additionally, the pharmacodynamic profiles of these drugs overlap with the receptor mechanisms known to cause SD.	**√**

ROR, reporting odds ratio (a measure of disproportionality).

√ Relevant criterium is fulfilled based on pharmacovigilance data.

? Uncertainty in fulfillment of relevant criterium.

## 4 Discussion

To the best of our knowledge, the current pharmacovigilance-pharmacodynamic (PV-PD) study is the first to investigate the AAP-SD associations using FAERS. There has been limited and inconsistent evidence comparing antipsychotics to each other for SD-related side effects. Based on our analysis of extensive real-world data, we believe that our study can provide significant additional insight. SD frequently occurs during short- and long-term treatment with AAPs; regrettably, clinical psychiatrists do not accord it the necessary attention it warrants. In this study, we leveraged a real-world AE reporting database to investigate signals of SD among patients receiving commonly prescribed AAPs, hoping to contribute additional insights to this subject. PV-PD studies represent an innovative approach to establishing a correlation between the binding affinities of drugs for their pharmacological targets and the associated reporting risks for AEs observed within an extensive pharmacovigilance database (Nguyen et al., 2017). To illustrate, this approach has recently been employed to explore the etiology of AEs like antidepressant-induced hyponatremia (Mazhar et al., 2019), manic switch (Çiray et al., 2021), antipsychotics-related pneumonia (Cepaityte et al., 2021), obsessive-compulsive disorder (Burk et al., 2023), and impulse control disorders (Fusaroli et al., 2022). Our findings could contribute greatly to the understanding of antipsychotic medications and SD-related side effects and help develop safer and more effective treatments for patients.

Aripiprazole has been consistently considered a sex-sparing molecule, reporting much less sexual disturbance than other widely used antipsychotics like risperidone, olanzapine, or quetiapine (Hanssens et al., 2008). Nevertheless, the current study unveiled a remarkable revelation. That aripiprazole was among the top agents for sexual functioning complaints, with high disproportionate reporting of SD. We found that such results were mainly driven by the higher ROR values for hypersexuality-related PTs including “compulsive sexual behavior”, “hypersexuality” etc. Coherently, aripiprazole has been reported to directly contribute to hypersexual behavior in several cases (Schlachetzki and Langosch, 2008; Cheon et al., 2013; Vrignaud et al., 2014; Bulbena-Cabré and Bulbena, 2016; Kozian, 2019). For example, a 36-year-old female patient with schizophrenia, been prescribed aripiprazole 20 mg/day and fluoxetine 40 mg/day, exhibited hypersexuality by engaging in masturbation and sexual fantasies, and frequently used online pornography. Interestingly, her increased sexual urges and activities rapidly subsided within days after discontinuing aripiprazole (Cheon et al., 2013). In a recent study investigating the association between antipsychotics and obsessive-compulsive disorder/obsessive-compulsive symptoms (OCD/OCS), researchers found aripiprazole was observed to have a significant signal for OCD/OCS as compared to all other antipsychotics (Burk et al., 2023). Aripiprazole-induced OCD/OCS was often co-reported with compulsive sexual behaviors and hypersexuality. In addition, brexpiprazole, and cariprazine were also found to be among the top drugs associated with reports of OCD/OCS. A study by Fusaroli et al. (2022) found aripiprazole had the strongest association with drug-induced ICDs, followed by brexpiprazole, and cariprazine. The ICD events analyzed here included “compulsive sexual behavior”, “hypersexuality”, “excessive masturbation” and “libido increased”, which were included in the hypersexuality-related PT terms subgroup in our analysis. In line with these previous findings, we also found aripiprazole was observed with a significant signal in reporting hypersexuality, as well as brexpiprazole and cariprazine.

As indicated in several studies, paliperidone could be labeled as a sexolytic agent, due to its negative impact on sexual functioning (Montejo et al., 2021; Jannini et al., 2022). However, there is limited availability of reports regarding the impact of paliperidone on sexual functioning, and their findings are inconsistent. A comprehensive Cochrane review, encompassing five short-term (<12 weeks) randomized placebo-controlled trials with a total of 2215 participants, concluded that there were no significant adverse effects on sexual functioning linked to paliperidone palmitate (Nussbaum and Stroup, 2012). Likewise, another meta-analysis found minimal evidence in the association (Harrington and English, 2010). Nevertheless, correlation between paliperidone and SD still has been reported in some studies. A young male began to experience erectile dysfunction and retrograde ejaculation after 3 months of paliperidone palmitate treatment. The symptoms ameliorated and eventually disappeared after reducing the dosage and discontinuing the paliperidone (Madan et al., 2018). An open-label, randomized trial by Potkin et al. (2017) found the odds of SD were significantly lower in the aripiprazole group compared with the paliperidone palmitate group (*p* = 0.0012). The phenomenon was even obvious in adult patients aged 35 years or younger (16.1% vs70.0%). In our study, paliperidone is presumed as the antipsychotic agent with robust associations with hyposexuality, erectile dysfunction, and ejaculatory dysfunction, which provides more information on paliperidone-induced SD.

As a new AAP that was approved by the FDA for the treatment of schizophrenia in 2009, the number of submitted reports in FAERS is notably lower compared to other AAPs, so we excluded it in further data mining. However, it is noteworthy that when looking solely at the signal analysis results, iloperidone exhibited strong signals for reporting retrograde ejaculation, ejaculation failure, and priapism, emphasizing the significance of exploring its connection with sexual functioning. A previous disproportionality analysis also found that iloperidone was observed to be significantly associated with priapism and SD (Subeesh et al., 2019). This may arise from iloperidone’s multifaceted antagonistic impact on various receptors, including alpha-adrenergic receptor blockade (resulting in priapism and erectile dysfunction), dopamine receptor antagonism (contributing to decreased libido), and histamine receptor binding (interfering with sexual arousal) (Park et al., 2012; Rodriguez-Cabezas et al., 2014), which may explain the results of the current study to some extent. Given the scarcity of previously established mechanisms regarding iloperidone-related SD, it is anticipated that future clinical research will delve deeper into this association, thereby augmenting the available evidence.

The main regression analyses revealed D2, D3, and muscarinic receptor occupancy showed a significant effect on the association of AAP-indued SD. However, when considering the activity of different AAPs on receptors, only the 5-HT1A receptor showed a significant association. After accounting for activity and excluding potential AEs that might have an impact, no receptor demonstrated a significant association. AAPs can exert their effects with varying affinities on multiple receptor subtypes, which may partly explain the lack of statistical significance of the regression analysis. Serotonin and dopamine both play crucial roles in modulating sexual behavior. Dopamine, acting through the mesolimbic system, promotes sexual function (Agmo, 2003). Consequently, antipsychotics with potent anti-dopaminergic properties (such as risperidone) may hinder this process, resulting in hyposexuality. However, partial dopamine agonists like aripiprazole, brexpiprazole, and cariprazine may increase sexual behavior. Conversely, serotonin, potentially through alterations in receptors such as 5-HT1A may contribute to sexual disturbances (Esquivel-Franco et al., 2020). These receptors targeted by AAPs disrupt the delicate balance between stimulation and inhibition, impacting sexual arousal, orgasm, and ejaculation. Certainly, the specific pathways linking receptors to the AAPs-induced SD side effects remain unclear, and mixed effects on different receptors could be involved. Research indicates that the anticholinergic and alpha-adrenergic effects of certain antipsychotics can impact sexual function, which may include the inhibition of motivation and reward, increased sedation, and reduced peripheral vasodilatation (Knegtering et al., 2003; Haddad and Wieck, 2004). Specifically, the relationship between alpha-1 adrenergic receptor affinities of antipsychotics and the occurrence of priapism has been discussed in several previous studies (Andersohn et al., 2010; Subeesh et al., 2019; Misawa and Takeuchi, 2022). It is also reported that the occurrence of erectile problems associated with APs may account for endothelial dysfunction, which is characterized by decreased nitric oxide production due to the inhibition of endothelial nitric oxide synthase (Montes de Oca et al., 2005). Additionally, vasoconstriction resulting from beta-2 adrenergic effects may also contribute to this issue (Molinari et al., 2007). The foundation of human sexual function is complex, involving not only neurotransmitters and receptors but also psychological factors, sex hormones, etc. To conclude, the exact mechanism on the AP-induced SDs is complicated and necessitates more investigations.

The present study has several limitations. Firstly, the FAERS database, being a spontaneous reporting system, has inherent limitations, including inaccurate data, missing values, duplications, over-reporting, and under-reporting. Secondly, due to the substantial missing data, the current study did not account for factors that could potentially impact sexual function, such as body weight, comorbidities, and concomitant medications, which may skew our findings. Additionally, the PV-PD analysis could be influenced by various factors concerning pharmacodynamic data and disproportionality signals. Hence, caution is warranted when interpreting the identified receptor mechanism. Finally, it is important to note that this study solely examined frequently prescribed AAPs. Therefore, there is a need for future research to encompass a broader spectrum of medications to comprehensively assess the effects of psychotropic drugs on patients’ sexual functioning.

Despite its limitations, the spontaneous reporting system for adverse drug reactions remains a crucial tool for post-marketing drug safety surveillance. One of its most significant advantages is the “real-world” data it provides. Our research highlights a potentially significant yet often “taboo” issue in clinical practice with antipsychotic drugs. We particularly hope that clinicians will pay attention to the sexual issues in their clinical practice. It is essential to assess and understand patients’ sexual health concerns both before and after treatment, enabling quicker identification of the risk of treatment discontinuation due to poor SD tolerance. Monitoring for possible changes in sexual function during treatment is also crucial. If intolerable SD adverse effects occur, individualized management strategies should be considered, such as gradually reducing dosage, switching to medications with a better sexual profile, or, if necessary, using treatments for SD (Montejo et al., 2021).

## 5 Conclusion

Our findings indicate a safety signal regarding the reporting of SD-related AEs for AAPs. The present analysis indicates that the involvement of D2, D3, and 5-HT1A receptors might represent a plausible mechanism driving this relationship, yet no significant correlation persisted following sensitivity analyses. Clinicians must be aware of the SD induced by AAPs, conducting thorough and regular assessments of the sexual function of patients to effectively evaluate their tolerance to antipsychotic treatment and prevent potential treatment discontinuation. Given the inherent limitations of pharmacovigilance studies, it is preferable to validate these findings from FAERS through alternative prospective research studies that directly compare different antipsychotic agents.

## Data Availability

Publicly available datasets were analyzed in this study. This data can be found here: https://openvigil.sourceforge.net/.
