# Supplementary material for: The atypical antipsychotics and sexual dysfunction: a pharmacovigilance-pharmacodynamic study
Source: Front Pharmacol. 2024 Jul 9;15:1423075. doi: 10.3389/fphar.2024.1423075 (PMC11263075; doi:10.3389/fphar.2024.1423075)
Supplement: Supplementary file 1 [file DataSheet1.PDF]

Supplementary Material

The Atypical Antipsychotics and Sexual Dysfunction: A Pharmacovigilance-Pharmacodynamic Study

Table S1 MedDRA Preferred Terms for Sexual Dysfunction manifestation. We classify the PT terms with positive signals based on the manifestations of sexual dysfunction.

Sexual Dysfunction manifestation	MedDRA preferred terms
**Hypersexuality**	"compulsive sexual behavior," "hypersexuality," "excessive masturbation," "libido increased."
**Hyposexuality**	"sexual dysfunction," "male sexual dysfunction," "female sexual dysfunction," "libido disorder," "libido decreased," "loss of libido," "orgasm abnormal," "male orgasmic disorder," "female orgasmic disorder," "disturbance in sexual arousal," "anorgasmia."
**Erectile Dysfunction**	"erectile dysfunction," "priapism," "painful erection," "organic erectile dysfunction," "spontaneous penile erection."
**Ejaculatory Dysfunction**	"ejaculation disorder," "ejaculation failure," "ejaculation delayed," "retrograde ejaculation," "premature ejaculation."
**Symptoms not specified**	"infertility," "azoospermia," "oligospermia," "psychosexual disorder," "paraphilia," "exhibitionism," "gender dysphoria," "haematospermia."

Table S2 Signal strength for the sexual dysfunction AEs with atypical antipsychotics at PT level in FAERS. We calculated the reporting odds ratio (ROR) and information component (IC) as a measure of disproportionate reporting of sexual dysfunction. We recorded them as ROR (95% confidence interval) and IC (95% confidence interval).

HLGT	PT	Aripiprazole			Asenapine			Brexpiprazole			Cariprazine		
		N	ROR (95%CI)	IC (95%CI)	N	ROR (95%CI)	IC (95%CI)	N	ROR (95%CI)	IC (95%CI)	N	ROR (95%CI)	IC (95%CI)
**Sexual function and fertility disorders**	Sexual dysfunction	430	14.98 (13.59-16.53)	3.81 (3.45-4.2)	22	6.83 (4.49-10.39)	2.76 (1.82-4.2)	12	2.73 (1.55-4.81)	1.45 (0.82-2.55)	5	2.51 (1.04-6.03)	1.32 (0.55-3.19)
	Erectile dysfunction	116	1.82 (1.52-2.19)	0.86 (0.72-1.03)	-	-	-	-	-	-	9	2.17 (1.13-4.18)	1.12 (0.58-2.15)
	Priapism	81	7.81 (6.26-9.75)	2.92 (2.34-3.65)	4	3.55 (1.33-9.47)	1.83 (0.68-4.87)	-	-	-	-	-	-
	Infertility	23	5.81 (3.84-8.79)	2.51 (1.66-3.79)	-	-	-	-	-	-	-	-	-
	Ejaculation disorder	16	2.07 (1.26-3.38)	1.04 (0.64-1.7)	-	-	-	-	-	-	-	-	-
	Ejaculation delayed	15	10.35 (6.17-17.37)	3.31 (1.97-5.56)	-	-	-	-	-	-	-	-	-
	Male sexual dysfunction	13	8.52 (4.9-14.83)	3.04 (1.75-5.3)	-	-	-	-	-	-	-	-	-
	Ejaculation failure	12	2.23 (1.26-3.94)	1.15 (0.65-2.03)	-	-	-	-	-	-	-	-	-
	Azoospermia	5	4.24 (1.75-10.27)	2.06 (0.85-5)	-	-	-	-	-	-	-	-	-
	Oligospermia	5	17.35 (6.98-43.12)	4.02 (1.62-9.98)	-	-	-	-	-	-	-	-	-
**Sexual dysfunctions, disturbances and gender identity disorders**	Compulsive sexual behaviour	322	533.02 (435.9-651.78)	7.3 (5.97-8.92)	-	-	-	6	20.7 (9.25-46.34)	4.35 (1.94-9.74)	-	-	-
	Hypersexuality	204	56.08 (48.09-65.4)	5.49 (4.71-6.4)	-	-	-	8	12.36 (6.16-24.8)	3.62 (1.8-7.25)	6	20.44 (9.15-45.63)	4.34 (1.95-9.7)
	Libido increased	97	20.19 (16.4-24.86)	4.21 (3.42-5.19)	5	9.11 (3.78-21.94)	3.18 (1.32-7.66)	6	8.05 (3.61-17.96)	3 (1.35-6.7)	4	11.82 (4.43-31.57)	3.56 (1.33-9.5)
	Psychosexual disorder	78	145.8 (109.57-194)	6.47 (4.86-8.6)	-	-	-	-	-	-	-	-	-
	Libido decreased	55	1.75 (1.34-2.28)	0.8 (0.61-1.04)	-	-	-	-	-	-	5	2.43 (1.01-5.85)	1.28 (0.53-3.08)
	Loss of libido	49	2.45 (1.85-3.24)	1.28 (0.97-1.7)	6	2.83 (1.27-6.29)	1.5 (0.67-3.33)	-	-	-	-	-	-
	Disturbance in sexual arousal	28	12.27 (8.39-17.96)	3.55 (2.42-5.19)	-	-	-	-	-	-	-	-	-
	Excessive masturbation	15	31.74 (18.48-54.52)	4.8 (2.8-8.25)	-	-	-	-	-	-	-	-	-
	Libido disorder	15	4.22 (2.53-7.04)	2.06 (1.23-3.43)	-	-	-	-	-	-	-	-	-
	Anorgasmia	13	1.84 (1.06-3.17)	0.87 (0.5-1.5)	-	-	-	4	3.93 (1.47-10.49)	1.97 (0.74-5.26)	4	8.68 (3.25-23.17)	3.11 (1.17-8.31)
	Male orgasmic disorder	12	9.22 (5.18-16.43)	3.15 (1.77-5.62)	-	-	-	-	-	-	-	-	-
	Paraphilia	9	26.31 (13.18-52.5)	4.56 (2.29-9.11)	-	-	-	-	-	-	-	-	-
	Exhibitionism	8	59.24 (27.16-129.22)	5.55 (2.55-12.12)	-	-	-	-	-	-	-	-	-
	Gender dysphoria	8	45.57 (21.29-97.51)	5.25 (2.45-11.23)	-	-	-	-	-	-	-	-	-
	Female orgasmic disorder	3	3.47 (1.11-10.86)	1.78 (0.57-5.57)	-	-	-	-	-	-	-	-	-

Table S2 Continued

HLGT	PT	Clozapine			Iloperidone			Lurasidone			Olanzapine		
		N	ROR (95%CI)	IC (95%CI)	N	ROR (95%CI)	IC (95%CI)	N	ROR (95%CI)	IC (95%CI)	N	ROR (95%CI)	IC (95%CI)
**Sexual function and fertility disorders**	Sexual dysfunction	105	2.63 (2.17-3.19)	1.38 (1.14-1.67)	10	20.53 (11-38.31)	4.34 (2.33-8.1)	37	5.12 (3.71-7.08)	2.35 (1.7-3.24)	159	8.16 (6.97-9.55)	2.99 (2.56-3.5)
	Priapism	30	2.15 (1.5-3.08)	1.09 (0.76-1.57)	19	113.98 (72.2-179.94)	6.79 (4.3-10.72)	-	-	-	87	12.97 (10.47-16.07)	3.64 (2.94-4.52)
	Retrograde ejaculation	10	3.79 (2.03-7.1)	1.9 (1.02-3.55)	25	832.09 (552.77-1252.56)	9.58 (6.36-14.42)	-	-	-	17	13.28 (8.18-21.56)	3.68 (2.27-5.98)
	Ejaculation failure	-	-	-	10	117.31 (62.73-219.4)	6.85 (3.66-12.8)	-	-	-	-	-	-
	Ejaculation disorder	-	-	-	8	64.87 (32.28-130.35)	6 (2.99-12.06)	-	-	-	15	2.98 (1.79-4.96)	1.57 (0.94-2.61)
	Erectile dysfunction	-	-	-	8	7.86 (3.92-15.78)	2.96 (1.48-5.95)	-	-	-	132	3.21 (2.7-3.81)	1.67 (1.4-1.98)
	Painful erection	-	-	-	-	-	-	-	-	-	20	14.98 (9.57-23.44)	3.85 (2.46-6.02)
	Infertility	-	-	-	-	-	-	-	-	-	12	4.61 (2.61-8.15)	2.19 (1.24-3.87)
	Haematospermia	-	-	-	-	-	-		-	-	6	5.28 (2.36-11.82)	2.38 (1.06-5.33)
**Sexual dysfunctions, disturbances and gender identity disorders**	Excessive masturbation	3	4.33 (1.38-13.61)	2.09 (0.66-6.56)	-	-	-	-	-	-	-	-	-
	Libido decreased	-	-	-	3	5.92 (1.91-18.4)	2.56 (0.82-7.96)	27	3.62 (2.48-5.29)	1.85 (1.27-2.7)	31	1.51 (1.06-2.15)	0.59 (0.42-0.84)
	Orgasm abnormal	-	-	-	3	83.63 (26.83-260.7)	6.37 (2.04-19.87)	-	-	-	5	3.44 (1.42-8.29)	1.77 (0.73-4.27)
	Hypersexuality	-	-	-	-	-	-	11	10.33 (5.7-18.71)	3.35 (1.85-6.08)	13	4.44 (2.57-7.67)	2.14 (1.24-3.69)
	Loss of libido	-	-	-	-	-	-	10	2.1 (1.13-3.9)	1.07 (0.57-1.99)	33	2.53 (1.8-3.56)	1.33 (0.95-1.88)
	Libido increased	-	-	-	-	-	-	6	4.87 (2.18-10.87)	2.28 (1.02-5.08)	-	-	-
	Disturbance in sexual arousal	-	-	-	-	-	-	3	5.31 (1.71-16.52)	2.4 (0.77-7.47)	-	-	-
	Female orgasmic disorder	-	-	-	-	-	-	3	14.72 (4.7-46.04)	3.86 (1.23-12.07)	-	-	-
	Hypersexuality	-	-	-	-	-	-	11	10.33 (5.7-18.71)	3.35 (1.85-6.08)	-	-	-
	Libido disorder	-	-	-	-	-	-	-	-	-	10	4.31 (2.31-8.04)	2.09 (1.12-3.91)
	Paraphilia	-	-	-	-	-	-	-	-	-	3	12.52 (3.95-39.61)	3.6 (1.14-11.39)
	Psychosexual disorder	-	-	-	-	-	-	-	-	-	3	5.29 (1.69-16.55)	2.39 (0.76-7.46)

Table S2 Continued

HLGT	PT	Paliperidone			Quetiapine			Risperidone			Ziprasidone		
		N	ROR (95%CI)	IC (95%CI)	N	ROR (95%CI)	IC (95%CI)	N	ROR (95%CI)	IC (95%CI)	N	ROR (95%CI)	IC (95%CI)
**Sexual function and fertility disorders**	Erectile dysfunction	121	6.35 (5.31-7.6)	2.65 (2.21-3.17)	206	2.94 (2.56-3.37)	1.54 (1.34-1.77)	179	2.69 (2.32-3.11)	1.41 (1.22-1.64)	38	3.99 (2.9-5.49)	1.99 (1.45-2.74)
	Sexual dysfunction	80	8.75 (7.01-10.91)	3.11 (2.49-3.88)	143	4.27 (3.62-5.04)	2.07 (1.75-2.44)	153	4.83 (4.11-5.66)	2.24 (1.91-2.63)	26	5.69 (3.87-8.36)	2.5 (1.7-3.68)
	Priapism	29	9.08 (6.29-13.1)	3.17 (2.19-4.57)	203	18.66 (16.15-21.55)	4.1 (3.55-4.73)	159	15.09 (12.84-17.72)	3.82 (3.25-4.49)	52	33.37 (25.33-43.96)	5.02 (3.81-6.61)
	Ejaculation failure	23	14.33 (9.48-21.65)	3.81 (2.52-5.76)	-	-	-	22	3.91 (2.57-5.96)	1.95 (1.28-2.97)	3	3.71 (1.19-11.51)	1.89 (0.61-5.86)
	Retrograde ejaculation	16	26.89 (16.33-44.3)	4.7 (2.85-7.74)	10	4.5 (2.4-8.42)	2.14 (1.15-4.01)	13	6.2 (3.57-10.77)	2.6 (1.5-4.51)	-	-	-
	Ejaculation disorder	13	5.56 (3.22-9.6)	2.47 (1.43-4.26)	15	1.74 (1.05-2.9)	0.8 (0.48-1.32)	26	3.2 (2.18-4.72)	1.67 (1.13-2.45)	-	-	-
	Infertility	9	7.42 (3.85-14.32)	2.88 (1.49-5.55)	-	-	-	34	8.25 (5.86-11.63)	3 (2.13-4.22)	5	8.29 (3.44-19.98)	3.04 (1.26-7.33)
	Ejaculation delayed	6	13.37 (5.96-29.97)	3.72 (1.66-8.33)	-	-	-	-	-	-	-	-	-
	Azoospermia	4	11.21 (4.18-30.11)	3.47 (1.29-9.31)	-	-	-	-	-	-	-	-	-
	Male sexual dysfunction	4	8.48 (3.16-22.71)	3.07 (1.15-8.22)	7	4.06 (1.92-8.58)	2 (0.95-4.23)	-	-	-	-	-	-
	Painful erection	3	4.67 (1.5-14.52)	2.22 (0.71-6.89)	8	3.41 (1.7-6.87)	1.75 (0.87-3.53)	13	5.91 (3.4-10.25)	2.53 (1.46-4.39)	-	-	-
	Infertility female	-	-	-	17	4.94 (3.05-7.99)	2.28 (1.41-3.68)	-	-	-	-	-	-
	Organic erectile dysfunction	-	-	-	15	19 (11.19-32.26)	4.12 (2.43-7)	-	-	-	-	-	-
	Spontaneous penile erection	-	-	-	6	3.02 (1.35-6.77)	1.58 (0.71-3.54)	5	2.65 (1.1-6.4)	1.39 (0.58-3.37)	-	-	-
	Female sexual dysfunction	-	-	-	4	6.02 (2.23-16.27)	2.55 (0.94-6.9)	-	-	-	-	-	-
**Sexual dysfunctions, disturbances and gender identity disorders**	Loss of libido	44	7.3 (5.42-9.82)	2.85 (2.12-3.84)	36	1.61 (1.16-2.24)	0.69 (0.49-0.95)	52	2.47 (1.88-3.24)	1.29 (0.98-1.7)	-	-	-
	Libido decreased	40	4.21 (3.08-5.75)	2.07 (1.51-2.82)	69	1.98 (1.56-2.51)	0.98 (0.77-1.24)	84	2.54 (2.05-3.15)	1.33 (1.08-1.65)	-	-	-
	Anorgasmia	16	7.51 (4.59-12.3)	2.9 (1.77-4.74)	-	-	-	-	-	-	-	-	-
	Psychosexual disorder	13	52.12 (29.69-91.5)	5.61 (3.19-9.84)	7	7.37 (3.47-15.67)	2.84 (1.33-6.03)	19	22.51 (14.03-36.13)	4.35 (2.71-6.99)	-	-	-
	Hypersexuality	6	4.39 (1.97-9.79)	2.13 (0.95-4.74)	22	4.43 (2.91-6.77)	2.12 (1.39-3.24)	25	5.33 (3.58-7.92)	2.38 (1.6-3.55)	-	-	-
	Orgasm abnormal	5	7.4 (3.07-17.87)	2.88 (1.19-6.94)	-	-	-	7	2.98 (1.41-6.27)	1.56 (0.74-3.29)	-	-	-
	Libido disorder	4	3.69 (1.38-9.85)	1.88 (0.7-5.01)	-	-	-	-	-	-	-	-	-
	Premature ejaculation	3	5.2 (1.67-16.2)	2.37 (0.76-7.38)	-	-	-	-	-	-	-	-	-
	Libido increased	-	-	-	26	4.56 (3.09-6.73)	2.17 (1.47-3.19)	17	3.12 (1.93-5.04)	1.63 (1.01-2.63)	8	10.29 (5.13-20.64)	3.35 (1.67-6.72)
	Excessive masturbation	-	-	-	15	28.59 (16.64-49.11)	4.65 (2.71-7.99)	7	13.06 (6.09-28.02)	3.63 (1.69-7.78)	-	-	-
	Disturbance in sexual arousal	-	-	-	7	2.65 (1.26-5.59)	1.4 (0.66-2.94)	-	-	-	-	-	-
	Orgasmic sensation decreased	-	-	-	4	3.89 (1.44-10.45)	1.94 (0.72-5.21)	-	-	-	-	-	-
	Compulsive sexual behaviour	-	-	-	-	-	-	13	6.17 (3.56-10.72)	2.59 (1.49-4.5)	-	-	-
	Exhibitionism	-	-	-	-	-	-	5	31.94 (12.47-81.83)	4.8 (1.87-12.3)	-	-	-

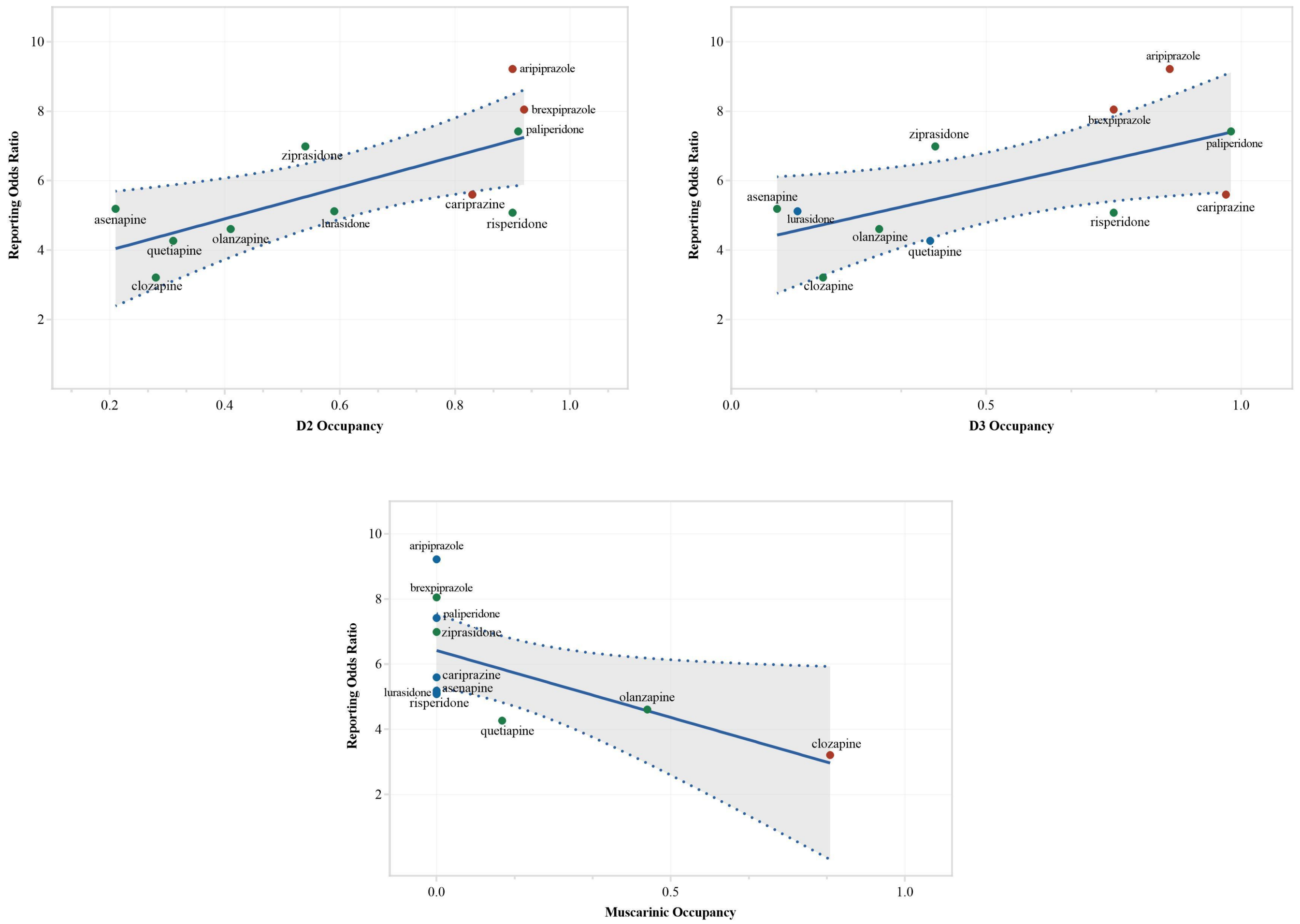

Figure S1. Association between activity on D2, D3 and Muscarinic receptor and reporting of sexual dysfunction with atypical antipsychotics – Main Analysis. Drugs were color-coded to show their activity: red indicates agonists (including partial agonists), green indicates antagonists, and blue indicates unavailable activity.
